# Emergency physician’s dispatch by a paramedic-staffed emergency medical communication centre: sensitivity, specificity and search for a reference standard

**DOI:** 10.1186/s13049-021-00844-y

**Published:** 2021-02-09

**Authors:** Victor Nathan Chappuis, Hélène Deham, Philippe Cottet, Birgit Andrea Gartner, François Pierre Sarasin, Marc Niquille, Laurent Suppan, Robert Larribau

**Affiliations:** 1 Division of Emergency Medicine, Department of Anaesthesiology, Clinical Pharmacology, Intensive Care and Emergency Medicine, Geneva University Hospital, Rue Gabrielle-Perret-Gentil 4, CH 1211 Geneva, Switzerland; 2 Division of Emergency Medicine, Department of Anaesthesiology, Clinical Pharmacology, Intensive Care and Emergency Medicine, Geneva University Hospital, Chemin du Petit-Bel-Air 2, CH 1226 Geneva, Thônex Switzerland

**Keywords:** Emergency medical dispatch, Paramedics, Emergency medical communication Centre, Emergency physician, Triage scale, Symptom based dispatch

## Abstract

**Background:**

Some emergency medical systems (EMS) use a dispatch centre where nurses or paramedics assess emergency calls and dispatch ambulances. Paramedics may also provide the first tier of care “in the field”, with the second tier being an Emergency Physician (EP).

In these systems, the appropriateness of the decision to dispatch an EP to the first line at the same time as the ambulance has not often been measured. The main objective of this study was to compare dispatching an EP as part of the first line emergency service with the severity of the patient’s condition. The secondary objective was to highlight the need for a recognized reference standard to compare performance analyses across EMS.

**Methods:**

This prospective observational study included all emergency calls received in Geneva’s dispatch centre between January 1st, 2016 and June 30th, 2019. Emergency medical dispatchers (EMD) assigned a level of risk to patients at the time of the initial call. Only the highest level of risk led to the dispatch of an EP. The severity of the patient’s condition observed in the field was measured using the National Advisory Committee for Aeronautics (NACA) scale. Two reference standards were proposed by dichotomizing the NACA scale. The first compared NACA≥4 with other conditions and the second compared NACA≥5 with other conditions. The level of risk identified during the initial call was then compared to the dichotomized NACA scales.

**Results:**

97′861 assessments were included. Overall prevalence of sending an EP as first line was 13.11, 95% CI [12.90–13.32], and second line was 2.94, 95% CI [2.84–3.05]. Including NACA≥4, prevalence was 21.41, 95% CI [21.15–21.67], sensitivity was 36.2, 95% CI [35.5–36.9] and specificity 93.2 95% CI [93–93.4]. The Area Under the Receiver-Operating Characteristics curve (AUROC) of 0.7507, 95% CI [0.74734–0.75397] was acceptable. Looking NACA≥5, prevalence was 3.09, 95% CI [2.98–3.20], sensitivity was 64.4, 95% CI [62.7–66.1] and specificity 88.5, 95% CI [88.3–88.7]. We found an excellent AUROC of 0.8229, 95% CI [0.81623–0.82950].

**Conclusion:**

The assessment by Geneva’s EMD has good specificity but low sensitivity for sending EPs. The dichotomy between immediate life-threatening and other emergencies could be a valid reference standard for future studies to measure the EP’s dispatching performance.

**Supplementary Information:**

The online version contains supplementary material available at 10.1186/s13049-021-00844-y.

## Background

Emergency Medical Dispatchers (EMD) are usually the first persons contacted by victims or by witnesses in a medical emergency [1, 2]. The role of the EMD is to decide whether it is appropriate to send emergency medical teams to the site and, if so, which team (Emergency Medical Technicians, Paramedics, Emergency Physician), and to assist the victim or witness until the emergency team arrives [3]. In some Emergency Medical Systems (EMS), the Emergency Medical Communication Centre (EMCC) is staffed by nurses or paramedics, who work as EMD. They answer emergency calls and dispatch ambulances using a Criteria-Based Dispatch system (CBD) [4].

EMS are often organized as a two-tiered system. Basic Life Support (BLS) teams are usually the first tier of response, the second one being composed of paramedics providing Advance Life Support (ALS). However, some countries in Europe uses paramedics as first tier responders (ALS-level 1) and Emergency Physicians (EP) as their second tier of response (ALS-level 2) to a medical emergency [5].

Only a few studies have compared EP presence on site and the effect on a patient’s outcome [6]. However, in specific situations such as cardiac arrest or trauma, authors have shown that the ability of an EP to provide more specific ALS (e.g. specific drugs administration, invasive ventilation, etc.) in life-threatening emergencies and to provide advanced decision-making [7], could *in fine* increase the patient’s survival rate [5, 8].

The accuracy of emergency medical dispatching, especially the dispatch of an EP at the same time as the ambulance, as the 1st line of response, is poorly understood. Only one article investigated the ability of an EMCC to detect the need for an on-site emergency physician [9]. The level of expertise provided by EP is usually expensive and rare [10]. The implementation of emergency medical dispatch must be measured in order to save available resources.

Measuring the performance of EMD implies the use of a recognised standard. Unfortunately, at present no such universally accepted reference-standard exists [11]. The National Advisory Committee for Aeronautics (NACA) scale is used in Switzerland [12] and other European countries [13] in pre-hospital medicine to assess a patient’s condition as encountered in the field and which is significantly correlated with the patient rate of survival [14–16]. Some countries in Europe use a dichotomized version of the NACA scale as their reference standard [17]. However, the NACA scale consists of 7 different levels and it is difficult to decide where or whether to dichotomize any of the different levels.

The main objective of this study was therefore to carry out a performance evaluation by comparing the decision to send an EP at the same time as the ambulance (as a 1st line of response) to the severity of the patient’s condition as observed in the field. The secondary objective was to emphasize the need for a valid reference standard in order to conduct further performance analysis of EMCCs.

## Materials and methods

This report follows the STAndards for Reporting of Diagnostic accuracy studies (STARD) statement guidelines for reporting diagnostic studies [18].

### Settings

The canton of Geneva, which covers an area of 282.48 km^2^, is essentially an urban canton with a population of 501′748 in 2018. 21% of the resident population was under the age of 20, 16.5% were over 64 and 51.5% were women. In addition to the resident population, about 100′000 cross-border workers commute daily from France or from neighbouring cantons to work in Geneva [19].

#### Geneva’s EMCC

Geneva’s single EMCC receives all emergency calls for the canton, handling over 68′000 calls per year. It is staffed by registered nurses or certified paramedics, with at least 5 years field experience. Since early 2013, Geneva’s EMD handle all calls from the beginning (interview) to the end (dispatching), evaluating situations with a Symptom-Based Dispatch (SBD) system.

#### Symptom-based dispatch (SBD) system

Unlike most EMCCs in the world, which use either a Criteria-Based Dispatch system (CBD) or Medical Priority Dispatch System (MPDS), Geneva’s EMCC has developed its own emergency medical dispatch system.

After assessing the state of consciousness and quality of breathing (with the aim of quickly identifying cardiac arrest), the EMD must select the most relevant symptom from a list of 53 symptoms adapted from the Swiss Emergency Triage Scale (SETS).

Once the symptoms have been assessed, Geneva’s EMD must then determine one of the five triage levels on a sorting scale, adapted from the SETS. Although, the SETS is composed of four main triage levels, SETS Level 1 (the most severe) was split into two (Level 1-A & Level 1-B). The difference between these two levels being that although the ambulance is dispatched with lights and sirens in both cases, an EP is only required for Level 1-A. If a more specific dispatch protocol for the assessment of the symptom exists (i.e. symptoms with an *), they must then choose a single determinant from those in the list to determine the level of triage. If there is no specific dispatch protocol, they will select the appropriate level of triage amongst the five existing levels.

Table 1 shows the correspondence between the five SETS triage levels and the three levels of departure priorities. In Geneva, only the first SETS level (level 1-A) leads to the simultaneous sending of an ambulance and an EP as a first response tier. An EP is usually sent simultaneously with the ambulance for three specific paediatric symptoms in children under 6 years of age and in these cases the triage level is therefore always level 1-A.

**Table 1:** Symptoms-Based Dispatch (SBD) priorities according to the Swiss Emergency Triage Scale (SETS)

SBD priorities	Swiss Emergency Triage Scale (SETS) (adapted)	
Priority 1 – Medicalized (P1-M)	Level 1-A	Immediately life-threatening situation, physician in the field necessary
Priority 1	Level 1-B	Immediately life-threatening situation, physician in the field not necessary
Priority 2	Level 2	Potentially life-threatening situation
Priority 3	Level 3	Stable situation (delayed departure)
	Level 4	Non-urgent situation (delayed departure)

#### Geneva’s emergency medical system (EMS)

In Geneva’s two-tier EMS system, paramedics staffing the ambulances are the first level of response and they have about thirty official protocols for the autonomous treatment of all symptoms. They can set up intravenous or intraosseous access, administer emergency medication, and perform all advanced cardiopulmonary resuscitation measures except endotracheal intubation. The second level of response is the pre-hospital EP who may be sent on site (by ground or helicopter) simultaneously with the ambulance, or later at the request of the paramedics. The EP dispatched by the EMD cannot reject the dispatch. There’s also a third tier, which is a senior physician, but this senior physician is only dispatched to the second line following a request from the team on the site.

### Reference standard

On site, the pre-hospital EP (if present) or the paramedic, assesses the patient’s condition according to the NACA scale [14], and informs the EMCC before leaving the site of intervention. As shown in Table 2, the NACA scale is a 7-level symptomatic scale, each level corresponding to a different level severity encountered on site. We separated level 7 into two in order to distinguish between a deceased patient, for whom there was no attempt at resuscitation (NACA 7 no-res), and a patient for whom resuscitation was attempted (NACA 7 res).

**Table 2:** Modified NACA scale dichotomized in 2 groups, as used in Geneva’s SBD system

	National Advisory Committee for Aeronautics (NACA) levels	
Symptoms usually not requiring an emergency physician on site	NACA 0	No injury or disease
	NACA 1	Injury/disease without any need for acute physician care
	NACA 2	Injury/disease requiring examination and therapy by a physician, but hospital admission is not indicated
	NACA 3	Injury/disease without threat of life but requiring hospital admission
	NACA 7 no res	Lethal injury or disease without resuscitation attempted
+/−	NACA 4	Injury/disease which can possibly lead to deterioration of vital signs
Symptoms usually requiring an emergency physician on site	NACA 5	Injury/disease with acute threat of life
	NACA 6	Injury/disease transported after an out-of-hospital cardiac arrest
	NACA 7 res	Lethal injuries or disease with resuscitation attempted (without transportation)

In Geneva’s SBD, a NACA ≥4 usually requires an EP on site. Main exceptions are: i) “*stroke (or suspicion)”* as main symptom tagged as NACA = 4, and ii) NACA 7 no-res (no attempt to resuscitate) where an EP is usually not necessary. A NACA < 4 does not normally require an EP on site. If the ambulance team arriving on site observes a NACA ≥4 and an EP was not dispatched simultaneously by the EMCC, the pre-hospital EP can then be sent by the EMCC as the second-line of response. For life-threatening emergencies (NACA ≥5), an EP should be dispatched on site to provide level 2 ALS.

### Selection of participants

Data sources from all emergency calls are collected in Geneva’s EMCC Computer-Aided Dispatch (CAD) software system. Inter-hospital transfers were excluded. In this study, we included all primary assessments for which a main symptom, a dispatch priority and a NACA scale were defined during the emergency call. Since the SBD system is specific to the Geneva EMCC, we decided to use a convenience sample, rather than a sample size calculation.

### Study design

This was a prospective observational study including data collected between 1st January 2016 and 30th June 2019.

First, measure the prevalence of the main symptoms presented by a victim having called Geneva’s EMCC and requiring emergency medical assistance.

Second, type of dispatch as defined during the initial call using Geneva’s SBD system on the basis of each reported symptom. Type of dispatch has been dichotomized into two groups: i) paramedics and emergency physician as 1st line response (Priority 1 – Medicalised), and ii) paramedics only as 1st line response (all other priorities).

Third, the NACA scale as observed in the field was identified for each symptom. This NACA scale has also been dichotomized into two groups: i) situations requiring an EP on site, and ii) no EP required on site.

Fourth, for each of the 53 symptoms defined during the original call, the “diagnostic test” of dispatch was compared to two proposed reference standards, as shown in Table 3:

- Proposed Reference Standard 1 (RS-1):
  - For situations NACA ≥4, an EP was usually required on site (= true positive).
  - For situations NACA < 4 and exceptions, no EP was required on site (= true negative).
- Proposed Reference Standard 2 (RS-2):
  - For situations NACA ≥5, an EP was necessarily required on site.
  - For situations NACA < 5, no EP was required on site.

**Table 3:** Contingency table for the “diagnostic test”

	Reference Standard (dichotomized NACA on-site)		
	*RS-1*	NACA 4, 5, 6, 7 res	NACA 0, 1, 2, 3, 7 no res
	*RS-2*	NACA 5, 6, 7 res	NACA 0, 1, 2, 3, 4, 7 no res
**EP dispatched as 1st line response**		True Positive	False Positive
**No EP dispatched as 1st line response**		False Negative	True Negative

The NACA scale measured by EP (if present) or paramedics for each symptom encountered in the field was considered as the reference standard depending on the severity associated with this symptom. The level of severity associated which each symptom, defined as the prevalence of a severe condition as observed in the field by the paramedics, was measured to interpret the predictive values. Sensitivity, specificity, Positive Predictive Value (PPV) & Negative Predictive Value (NPV) were calculated for each of the 53 symptoms. Over triage was defined as the proportion of an EP dispatch with an in the field NACA scale < 4 or < 5 (1 – *positive predictive value)*, and under triage was defined as the proportion of dispatch without an EP with an in the field NACA scale > 3 or > 4 (1 – *negative predictive value*).

A well performing dispatch system should not send an EP when it is not necessary, and therefore we were interested in all symptoms for which the specificity of sending an EP was lower than 50%.

Some situations may require the presence of an EP on site, even though this was not identified at the moment of dispatch (1st-line). To detect these situations, we analysed the ambulance team’s request for an EP (2nd-line dispatch). We then measured the ratio of 2nd-line dispatch over 1st line dispatch and looked at all symptoms for which the ratio was 2 or higher.

### Statistical analysis

The CSV file containing the data from the Geneva SBD system was imported into the STATA® 14.2 software (StataCorp®, College Station, TX USA).

AUROC, prevalence, sensitivity, specificity, predictive values and their 95% confidence interval calculations, as well as all descriptive statistics calculations, were performed using STATA® 14.2 software.

## Results

**Fig. 1:**
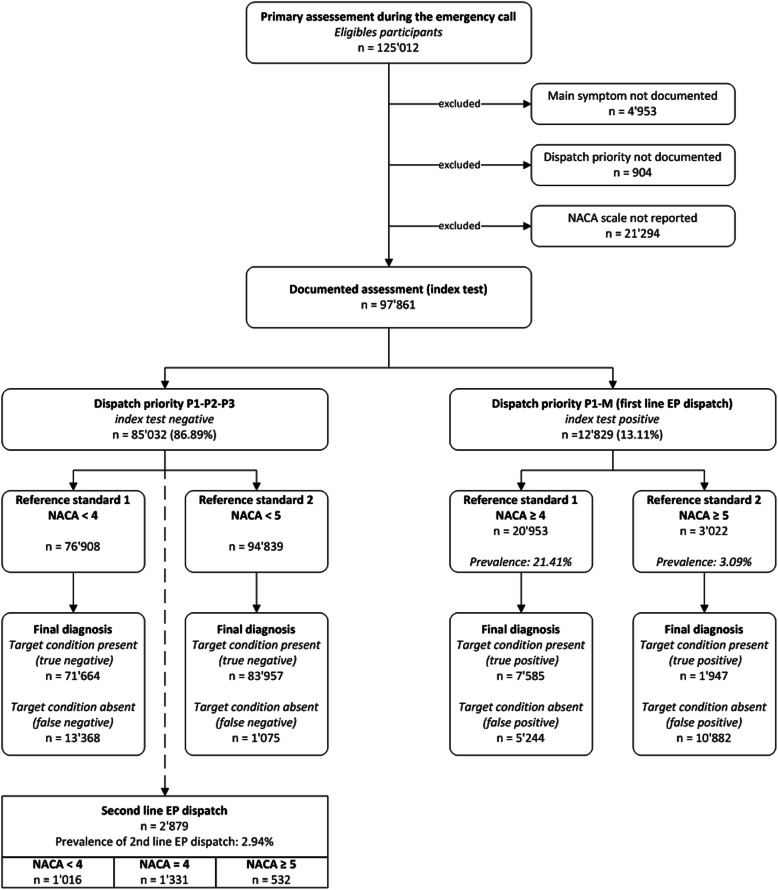
Flow chart of the study participants

During the study period, 125′012 primary evaluations were performed by Geneva’s EMCC. 27′151 assessments (21.7%) where found not to be fully documented and were consequently not included. Therefore, this study included 97′861 fully documented assessments. Figure 1 Flow chart of the study participants.

The prevalence of sending an EP as 1st-line was 13.11, 95% CI [12.90–13.32] and for sending a second-line EP, was 2.94, 95% CI [2.84–3.04]. The overall prevalence of NACA ≥5 observed in the field was 3.09, 95% CI [2.98–3.20] and NACA ≥4 was 21.41, 95% CI [21.15–21.67].

The prevalence of main symptoms (see Additional file 1) encountered is heterogeneous. The most common symptoms (> 5% of all evaluations) were “*Trauma of a limb*”, “*Dyspnea / shortness of breath*”, “*Chest pain*” and “*Cranio-cerebral trauma*”. Less common symptoms (< 0.05% of all evaluations) were “*Polytrauma or suspicion*”, “*Hypothermia*”, “*Electrocution*”, “*Bites*” and “*Diving accident*”. Additional file 2 is sorted in ascending order depending on the prevalence of NACA ≥5 situations. This prevalence differs significantly depending on the main symptom it is associated with. This Additional file 2 links the prevalence of the priority dispatch of an EP (i.e. 1st-line and 2nd-line) with the prevalence of the NACA scale observed on site (i.e. *RS-1* (NACA ≥4); *RS-2* (NACA ≥5)) for each of the 53 symptoms.

**Fig. 2:**
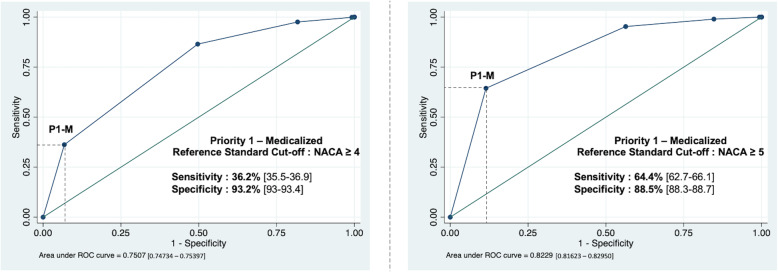
Receiver Operating Characteristic (ROC) curves of Priority Dispatch Level “Priority 1 – Medicalised” to predict NACA ≥4 (GS-1) and NACA ≥5 (GS-2)

Figure 2 shows the ROC curve of the dispatch performance, respectively for reference-standards *RS-1* and *RS-2*. Overall dispatch performance is displayed by the AUROC. For *RS-2 (NACA ≥ 5)*, we found an AUROC of 0.8229, 95% CI [0.81623–0.82950], and for *RS-1 (NACA ≥ 4)* an AUROC of 0.7507, 95% CI [0.74734–0.75397] [20].

Considering all assessments, Geneva’s EMD operate with a sensitivity of 36.2, 95% CI [35.5–36.9%] and a specificity of 93.2, 95% CI [93.0–93.4%] if *RS-1* is used as the reference standard. Still using *RS-1*, the under-triage rate is 15.7, 95% CI [15.5–16.0] and the over-triage rate is 41, 95% CI [40.0–41.7]. If RS-2 is used as the reference standard, we found a sensitivity of 64.4, 95% CI [62.7–66.1] and a specificity of 88.5, 95% CI [88.3–88.7]. The under-triage rate is 1.3, 95% CI [1.2–1.3] and the over-triage rate is 84.8, 95% CI [84.2–85.4] when using *RS-2*.

The sensitivity, specificity, positive and negative predictive value for each of the 53 symptoms as well as the over and under-triage rates with their respective 95% confidence intervals for *RS-1 (NACA ≥ 4)* can be found in *Additional file 3* and for *RS-2 (NACA ≥* 5) in Additional file 4.

Table 4 details the six symptoms that have a specificity lower than 50% for NACA ≥4. The symptom with the lowest specificity was *“cardiac arrest or death”* (19.6% [16.0–23.5%]).

**Table 4:** Symptoms with specificity < 50% (RS-1 NACA ≥4)

Symptoms	Specificity [95% CI]		Sensitivity [95% CI]	
Cardiac arrest or death*	19.6%	[16–23.5%]	98.9%	[97.7–99.6%]
Seizure / febrile condition in children under 6 years	20.5%	[16.6–24.9%]	93.5%	[82.1–98.6%]
Polytrauma (or suspicion)	25%	[7.3–52.4%]	88.9%	[70.8–97.6%]
Newborn and infant evaluation	36.9%	[25.3–49.8%]	77.3%	[54.6–92.2%]
Choking*	45.2%	[35.4–55.3%]	82.6%	[68.6–92.2%]
Respiratory difficulty in children under 6 years	45.2%	[40.5–50%]	87.8%	[79.6–93.5%]

*Specific dispatch protocol for symptom assessment

Finally, Table 5 shows the twelve symptoms having a ratio > 2 of sending an EP as 2nd-line over 1st line, meaning that for these 12 symptoms, an EP was sent twice or more often as a backup rather than as 1st line. *“Alcoholic intoxication”* and *“Confusion hallucinations”* have the highest ratio for sending an EP as 2nd line over 1st line, respectively 12.5 and 10.89. It should be noted that for the symptom *“Social hospitalization”*, as the prevalence of 1st line was 0%, the ratio 2nd line/1st line was not calculable.

**Table 5:** Symptoms with a 2nd-line/1st-line ratio > 2

Symptoms	Ratio 2nd line / 1st line	Prev. 1st line	Prev. 2nd line
Social hospitalization	–	0.00%	2.34%
Alcoholic intoxication	12.00	0.22%	2.62%
Confusion / hallucination	10.78	0.54%	5.77%
Anxiety / depression	7.83	0.51%	4.01%
Unspecified malaise	5.55	0.67%	3.74%
Syncope / lipothymia	4.00	0.85%	3.41%
Back pain	3.33	0.21%	0.70%
Kidney pain	3.00	0.19%	0.58%
Pain / oedema of a limb	3.00	0.28%	0.85%
Genital or urinary involvement	2.50	0.19%	0.48%
Panic attack / suicidal ideation	2.28	2.13%	4.85%
Stroke (or suspicion)	2.06	1.31%	2.69%

## Discussion

The EP is usually a scarce and expensive resource. Therefore, this EP must be assigned to the 1st line in the most efficient manner. In a high-performance dispatch system, we expected that a severe condition requiring an EP on site should be categorized as *Priority 1 – Medicalised (P1-M),* which would result in sending an EP at the same time as the ambulance as a 1st line response. It appears that Geneva’s EMD regulate with good specificity, but low sensitivity. In our system, this poor sensitivity could be acceptable for NACA ≥4 situations, because ambulances are staffed with paramedics providing ALS as the EMS 1st line response.

Leopardi and al [9]. found approximately the same specificity level (83%) in the only study we found reviewing “the ability of a dispatch centre [ …] to detect pre-hospital need for physician interventions”. Their sensitivity (78%) is better than ours with NACA ≥4 (36%), but at the cost of a higher over-triage rate (64% vs 41%). However, the reference standard used in this Italian study is not exactly comparable to the one we used.

In as much that there is no shared reference standard for assessing the severity of a patient’s condition, it is somewhat difficult to compare dispatch performance involving different EMCC. Immediate life threatening situations, corresponding to a NACA ≥5 level, seem to be present in most systems, for example when Ball and al [21]. look at “time-critical” situations in Australia. We found that the prevalence of NACA ≥5 (*n* = 3.1%) in our study is similar to the prevalence of “time-critical” situations found in Australia (*n* = 3.3%). Furthermore, Dami and al [12], .whose study covers a nearby canton with a comparable population, have a 14% (vs 21% in our study) prevalence of NACA ≥4 in their EMCC.

We find that Physician-based Helicopter EMS (P-HEMS) mainly take care of patients with NACA ≥4 [22, 23]. However, NACA 4 is defined as a condition that may possibly lead to deterioration of vital signs, and not as a situation with an acute threat to life (NACA definition ≥5). For acute threats to life, the added value of the EP seems to be unproven [24], but could provide added value in a few specific situations [25]. Where a patient’s conditions may lead to a deterioration of vital signs (NACA 4), this is less obvious, as no specific medical acts are carried out in the field [26]. There is only a higher risk of deterioration of vital signs, which could be monitored by paramedics [27]. These observations suggest that the NACA scale measurements are quite subjective, especially when the NACA is low [28]. Dichotomization at the NACA level ≥ 4 may therefore not be sufficiently objective. We suggest therefore that life-threatening emergencies, for example NACA ≥5, should be considered as a valid reference standard to conduct performance studies when dispatching an EP (or the highest level of response available) in order to compare different EMCC.

It is possible to estimate triage performance for each symptom defined during the emergency call by measuring the sensitivities, specificities and predictive values for each of them (Additional file 3 and Additional file 4). In this way, it is possible to identify the symptoms for which the EP’s dispatch needs to be improved (e.g. by introducing specific assessment protocols) [29]. Moreover, by constructing ROC curves for each symptom, it is also possible to improve the accuracy of the discriminating questions for each symptom and thus improve the quality of triage. In Table 4, it appears that for paediatric patients (“*Seizure / febrile condition in children under 6 years*”, “*Respiratory difficulty in children under 6 years*” and “*Newborn and infant evaluation*”), the rule tends to be the sending of an EP quickly regardless of the severity of the patient, resulting in poor specificity for these symptoms. Given the relatively low prevalence of serious situations observed (respectively 13.11, 18.22 and 25.29%), this rule should probably be reconsidered. The very low prevalence (0.04%) of “*Polytrauma (or suspicion)*” observed does not allow conclusions to be drawn on the observed specificity value. For patients whose main symptom is “*Choking*”, the success of the Heimlich manoeuvre provided by witnesses present at the event between the initial call to the EMCC and the arrival of the ambulance on site, reduces the number of serious situations observed. This may explain the low specificity measured for this symptom. During the initial call, it is clearly difficult for the dispatcher to fully assess situations where reanimation will be attempted or not on patients with “*Cardiac arrest or death”* as the main symptom, and this might explain why they often send an EP as 1st line response in order to minimize under-triage, resulting in a poor specificity.

Situations where the rate of 2nd line dispatch of an EP is higher than the 1st line (see Table 5) could potentially be explained in two ways: either there is a failure in the initial dispatch of an EP, or an EP is necessary even though the patient’s condition is not severe. Indeed, when an EP was sent to the second line, there were still more than a third of NACA < 4 found in the field by this EP (Fig. 2)*.* Decision making (e.g. committing a patient to a psychiatric ward) could be one of the main reasons why an EP is requested for psychiatric situations (“*Social hospitalization*”; “*Confusion/hallucination*”; “*Anxiety/depression*”; “*Panic attack/suicidal ideation*”), even though the patient’s condition is not severe (prevalence of life threatening situations < 1% for all psychiatric situations). The EMD probably miss serious situations in certain cases such as “*Kidney pain*”; “*Back pain*”; “*Syncope/lipothymia*”; “*Pain/oedema of a limb*”, “*Genital or urinary involvement*” and “*Alcoholic intoxication*”. It is difficult to evaluate the severity of these conditions without specific protocols. “*Unspecified malaise*” means that it was not possible to accurately characterize the patient’s main complaint, making it even more difficult to assess its severity.

The Geneva EMS, decided that NACA = 4 related to a stroke (or suspicion of), does not require an EP on site even if it is a situation that can lead to the rapid deterioration of vital signs. There is, however, a high rate of second-line EP dispatch for this situation. It is likely that life-threatening emergencies for “*Stroke (or suspicion)*” are not well detected in the initial call, which constitutes a “simplification bias”. Finally, we noticed that all symptoms with a high 2nd-line dispatch rate presented in Table 5 lack dispatch protocols (no *), which could potentially highlight their added value.

### Limitations and strengths

There are several limitations to our study, due to the fact that it was an observational and mono-centric study. Furthermore, we cannot exclude a selection bias as no documentation was found for approximately 22% of all primary assessments. Finally, the SBD system derived from the SETS is specific to Geneva’s EMCC, and Geneva’s EMS mobilizes two (or three) advanced levels of emergency care, including an EP. Few EMS are therefore comparable to the Geneva system, and this limits the generalisation of the results of this study.

However, this is a prospective study, conducted on a large sample of telephone assessments, which measures the accuracy of dispatching the emergency physician by paramedics or nurses. This study highlights areas for improvement which may require future research, showing the need to detail and analyse assessments for each of the symptoms identified during the call, not just to perform overall assessments. This study also shows the importance of defining a reliable reference-standard for comparing emergency dispatch systems.

## Conclusion

Paramedics and nurses working as EMD in an EMCC send the EP with a good specificity, especially for life-threatening emergencies, but their sensitivity remains low. The prevalence of life-threatening emergencies is very heterogeneous depending on the main symptom. For each of these main symptoms, we also observe a very large heterogeneity in the values of sensitivities and specificities measured. To improve the quality of the EP’s dispatch, it is therefore essential to measure these values for each of the main symptoms identified during the emergency call.

The performance of the emergency physician’s dispatch should be comparable to a universally agreed reference standard. The “dichotomized NACA scale” reference standard remains imperfect. An immediate life-threatening emergency (i.e. NACA ≥5) seems to be the most objective reference standard for comparing the accuracy of the EP’s dispatch between EMCCs. Using the same patient’s assessment tool during the emergency call and when the emergency physician arrives in the field would most likely be an even better reference standard. Future studies are needed to validate these proposed reference standards.

## Supplementary Information

**Additional file 1.** Prevalence of main symptoms, NACA ≥4 & NACA ≥5, 1st & 2nd- line dispatch. This table shows the prevalence of the main symptoms identified during the call, the prevalence respectively of NACA ≥4 and NACA ≥5 found on site for each symptom, and the prevalence of 1st & 2nd line dispatch of the emergency physician for each symptom.

**Additional file 2 **Prevalence of NACA ≥4 & NACA ≥5 associated with the prevalence of sending an EP as 1st & 2nd line. This figure links the prevalence of the priority dispatch of an EP (i.e. 1st-line and 2nd-line) with the prevalence of the NACA scale observed on site (i.e. *RS-1* (NACA ≥4); *RS-2* (NACA ≥5)) for each of the 53 symptoms.

**Additional file 3.** “Diagnostic test” for each symptom using NACA ≥4 as reference standard. This table shows the “diagnostic test” applied for each of the 53 symptoms, thus detailing the sensitivity, specificity, positive & negative predictive values, over-triage & under-triage with their respective 95% confidence intervals for each symptom when using NACA ≥4 as reference standard.

**Additional file 4.** “Diagnostic test” for each symptom using NACA ≥5 as reference standard. This table shows the “diagnostic test” applied for each of the 53 symptoms, thus detailing the sensitivity, specificity, positive & negative predictive values, over-triage & under-triage with their respective 95% confidence intervals for each symptom when using NACA ≥5 as reference standard.

## Data Availability

The datasets used and/or analysed in the present study are available from the corresponding author on reasonable request.

## Abbreviations

ALS: Advance Life Support
AUROC: Area Under the ROC Curve
BLS: Basic Life Support
CAD: Computer Aided Dispatch
CBD: Criteria-Based Dispatch system
EMCC: Emergency Medical Communication Centre
EMD: Emergency Medical Dispatchers
EMS: Emergency Medical Systems
EP: Emergency Physician
MPDS: Medical Priority Dispatch System
NACA: National Advisory Committee for Aeronautics
NPV: Negative Predictive Value
P1-M: Priority 1 – Medicalised
PPV: Positive Predictive Value
ROC: Receiver Operating Characteristic curve
RS-1: Reference-Standard 1 (NACA ≥4)
RS-2: Reference Standard 2 (NACA ≥5)
SBD: Symptoms-Based Dispatch system
SETS: Swiss Emergency Triage Scale
