# Supplementary material for: Emergency physician’s dispatch by a paramedic-staffed emergency medical communication centre: sensitivity, specificity and search for a reference standard
Source: Scand J Trauma Resusc Emerg Med. 2021 Feb 9;29:31. doi: 10.1186/s13049-021-00844-y (PMC7871575; doi:10.1186/s13049-021-00844-y)
Supplement: Supplementary file 1 — Additional file 1. Prevalence of main symptoms, NACA ≥4 & NACA ≥5, 1st & 2nd- line dispatch. This table shows the prevalence of the main symptoms identified during the call, the prevalence respectively of NACA ≥4 and NACA ≥5 found on site for each symptom, and the prevalence of 1st & 2nd line dispatch of the emergency physician for each symptom. [file 13049_2021_844_MOESM1_ESM.pdf]

Additional file #1

Prevalence of main symptoms, NACA ≥ 4 & NACA ≥ 5, 1st & 2nd- line dispatch

	PREVALENCE OF MAIN SYMPTOMS			PREVALENCE OF NACA ≥ 4				PREVALENCE OF NACA ≥ 5				PREVALENCE OF 1ST LEVEL ; 2ND LEVEL ; 1ST & 2ND LEVEL					
**Symptoms**	**#**	**Total**	**%**	**NACA < 4 & 7no rea**	**NACA ≥ 4**	**Total**	**% NACA ≥ 4**	**NACA<5 & 7no rea**	**NACA ≥ 5**	**Total**	**% NACA ≥ 5**	**1st level**	**% 1st level**	**2nd level**	**% 2nd level**	**Total**	**% 1st & 2nd level**
Kidney pain	517	97861	0.53%	499	18	517	3.48%	517	0	517	0.00%	1	0.19%	3	0.58%	517	0.77%
Anxiety / depression	1172	97861	1.20%	1125	47	1172	4.01%	1172	0	1172	0.00%	6	0.51%	47	4.01%	1172	4.52%
Oto-rhino-laryngological problems	249	97861	0.25%	231	18	249	7.23%	249	0	249	0.00%	2	0.80%	2	0.80%	249	1.61%
Bites	13	97861	0.01%	12	1	13	7.69%	13	0	13	0.00%	0	0.00%	0	0.00%	13	0.00%
Ophthalmological problems	61	97861	0.06%	56	5	61	8.20%	61	0	61	0.00%	0	0.00%	0	0.00%	61	0.00%
Hypothermia	36	97861	0.04%	27	9	36	25.00%	36	0	36	0.00%	5	13.89%	1	2.78%	36	16.67%
Genital or urinary involvement	1038	97861	1.06%	998	40	1038	3.85%	1036	2	1038	0.19%	2	0.19%	5	0.48%	1038	0.67%
Agitation / aggressiveness	1946	97861	1.99%	1749	197	1946	10.12%	1941	5	1946	0.26%	85	4.37%	138	7.09%	1946	11.46%
Panic attack / suicidal ideation	1878	97861	1.92%	1622	256	1878	13.63%	1873	5	1878	0.27%	40	2.13%	91	4.85%	1878	6.98%
Spinal trauma	1824	97861	1.86%	1698	126	1824	6.91%	1819	5	1824	0.27%	74	4.06%	26	1.43%	1824	5.48%
Confusion / hallucination	1680	97861	1.72%	1440	240	1680	14.29%	1675	5	1680	0.30%	9	0.54%	97	5.77%	1680	6.31%
Trauma of a limb	9830	97861	10.04%	9317	513	9830	5.22%	9797	33	9830	0.34%	81	0.82%	84	0.85%	9830	1.68%
Nausea, vomiting, diarrhoea	1219	97861	1.25%	1111	108	1219	8.86%	1214	5	1219	0.41%	12	0.98%	18	1.48%	1219	2.46%
Back pain	1428	97861	1.46%	1380	48	1428	3.36%	1422	6	1428	0.42%	3	0.21%	10	0.70%	1428	0.91%
Pain / oedema of a limb	1056	97861	1.08%	998	58	1056	5.49%	1051	5	1056	0.47%	3	0.28%	9	0.85%	1056	1.14%
Alcoholic intoxication	2749	97861	2.81%	2620	129	2749	4.69%	2734	15	2749	0.55%	6	0.22%	72	2.62%	2749	2.84%
Social hospitalization	299	97861	0.31%	287	12	299	4.01%	297	2	299	0.67%	0	0.00%	7	2.34%	299	2.34%
Abdominal pain (non-traumatic)*	4377	97861	4.47%	4072	305	4377	6.97%	4347	30	4377	0.69%	49	1.12%	53	1.21%	4377	2.33%
High blood pressure	923	97861	0.94%	687	236	923	25.57%	916	7	923	0.76%	68	7.37%	33	3.58%	923	10.94%
Ingestion, inhalation or exposure to a toxic	127	97861	0.13%	110	17	127	13.39%	126	1	127	0.79%	15	11.81%	11	8.66%	127	20.47%
Abdominal or pelvic trauma*	1097	97861	1.12%	1016	81	1097	7.38%	1088	9	1097	0.82%	45	4.10%	7	0.64%	1097	4.74%
Wounds	1677	97861	1.71%	1546	131	1677	7.81%	1663	14	1677	0.83%	51	3.04%	28	1.67%	1677	4.71%
Headache*	911	97861	0.93%	671	240	911	26.34%	903	8	911	0.88%	50	5.49%	9	0.99%	911	6.48%
Other	4715	97861	4.82%	4322	393	4715	8.34%	4673	42	4715	0.89%	88	1.87%	76	1.61%	4715	3.48%
Maxillofacial trauma	1929	97861	1.97%	1805	124	1929	6.43%	1911	18	1929	0.93%	26	1.35%	34	1.76%	1929	3.11%
Fever / flue-like condition	1269	97861	1.30%	1139	130	1269	10.24%	1255	14	1269	1.10%	27	2.13%	21	1.65%	1269	3.78%
Cranio-cerebral trauma	5652	97861	5.78%	5031	621	5652	10.99%	5579	73	5652	1.29%	244	4.32%	114	2.02%	5652	6.33%
Respiratory difficulty in children under 6 years	538	97861	0.55%	440	98	538	18.22%	531	7	538	1.30%	327	60.78%	2	0.37%	538	61.15%
Intoxication with drugs / overdose	2255	97861	2.30%	1713	542	2255	24.04%	2225	30	2255	1.33%	51	2.26%	90	3.99%	2255	6.25%
Unspecified malaise	4304	97861	4.40%	3706	598	4304	13.89%	4242	62	4304	1.44%	29	0.67%	161	3.74%	4304	4.41%
Chest trauma	654	97861	0.67%	570	84	654	12.84%	644	10	654	1.53%	43	6.57%	13	1.99%	654	8.56%
Stroke (or suspicion)	3897	97861	3.98%	1414	2483	3897	63.72%	3837	60	3897	1.54%	51	1.31%	105	2.69%	3897	4.00%
Syncope / lipothymia	3404	97861	3.48%	2971	433	3404	12.72%	3347	57	3404	1.67%	29	0.85%	116	3.41%	3404	4.26%
Seizure / febrile condition in children under 6 years	351	97861	0.36%	305	46	351	13.11%	344	7	351	1.99%	269	76.64%	0	0.00%	351	76.64%
Threat of childbirth / childbirth*	244	97861	0.25%	163	81	244	33.20%	239	5	244	2.05%	83	34.02%	5	2.05%	244	36.07%
Heart rhythm disorder	2243	97861	2.29%	1357	886	2243	39.50%	2188	55	2243	2.45%	524	23.36%	127	5.66%	2243	29.02%
Miscarriage, vaginal bleeding, pregnancy	241	97861	0.25%	166	75	241	31.12%	235	6	241	2.49%	15	6.22%	4	1.66%	241	7.88%
Allergies*	895	97861	0.91%	640	255	895	28.49%	872	23	895	2.57%	303	33.85%	24	2.68%	895	36.54%
Burns	132	97861	0.13%	108	24	132	18.18%	128	4	132	3.03%	40	30.30%	2	1.52%	132	31.82%
New born and infant evaluation	87	97861	0.09%	65	22	87	25.29%	84	3	87	3.45%	58	66.67%	0	0.00%	87	66.67%
Bleeding	1821	97861	1.86%	1368	453	1821	24.88%	1752	69	1821	3.79%	144	7.91%	27	1.48%	1821	9.39%
Hypoglycaemia / hyperglycaemias	421	97861	0.43%	339	82	421	19.48%	405	16	421	3.80%	9	2.14%	14	3.33%	421	5.46%
Chest pain*	6017	97861	6.15%	2578	3439	6017	57.15%	5773	244	6017	4.06%	3549	58.98%	222	3.69%	6017	62.67%
Convulsions / seizure*	1816	97861	1.86%	1339	477	1816	26.27%	1735	81	1816	4.46%	256	14.10%	106	5.84%	1816	19.93%
Arterial hypotension, shock	909	97861	0.93%	606	303	909	33.33%	862	47	909	5.17%	170	18.70%	53	5.83%	909	24.53%
Electrocution	35	97861	0.04%	27	8	35	22.86%	33	2	35	5.71%	14	40.00%	0	0.00%	35	40.00%
Person lying, without possibility to evaluate	4184	97861	4.28%	3321	863	4184	20.63%	3895	289	4184	6.91%	629	15.03%	218	5.21%	4184	20.24%
Dyspnoea / shortness of breath*	9043	97861	9.24%	5421	3622	9043	40.05%	8292	751	9043	8.30%	2721	30.09%	466	5.15%	9043	35.24%
Choking*	150	97861	0.15%	104	46	150	30.67%	135	15	150	10.00%	95	63.33%	1	0.67%	150	64.00%
Diving accident*	9	97861	0.01%	4	5	9	55.56%	8	1	9	11.11%	4	44.44%	0	0.00%	9	44.44%
Coma / disturbance of consciousness*	3475	97861	3.55%	2148	1327	3475	38.19%	3077	398	3475	11.45%	1461	42.04%	123	3.54%	3475	45.58%
Polytrauma (or suspicion)	43	97861	0.04%	16	27	43	62.79%	32	11	43	25.58%	36	83.72%	0	0.00%	43	83.72%
Cardiac arrest or death*	1021	97861	1.04%	450	571	1021	55.93%	556	465	1021	45.54%	927	90.79%	4	0.39%	1021	91.19%
**TOTAL**	**97861**	97861	100.00%	**76908**	**20953**	**97861**	**21.41%**	**94839**	**3022**	**97861**	**3.09%**	**12829**	**13.11%**	**2879**	**2.94%**	**97861**	**16.05%**
