# Supplementary figures and images for: Emergency physician’s dispatch by a paramedic-staffed emergency medical communication centre: sensitivity, specificity and search for a reference standard
Source: Scand J Trauma Resusc Emerg Med. 2021 Feb 9;29:31. doi: 10.1186/s13049-021-00844-y (PMC7871575; doi:10.1186/s13049-021-00844-y)

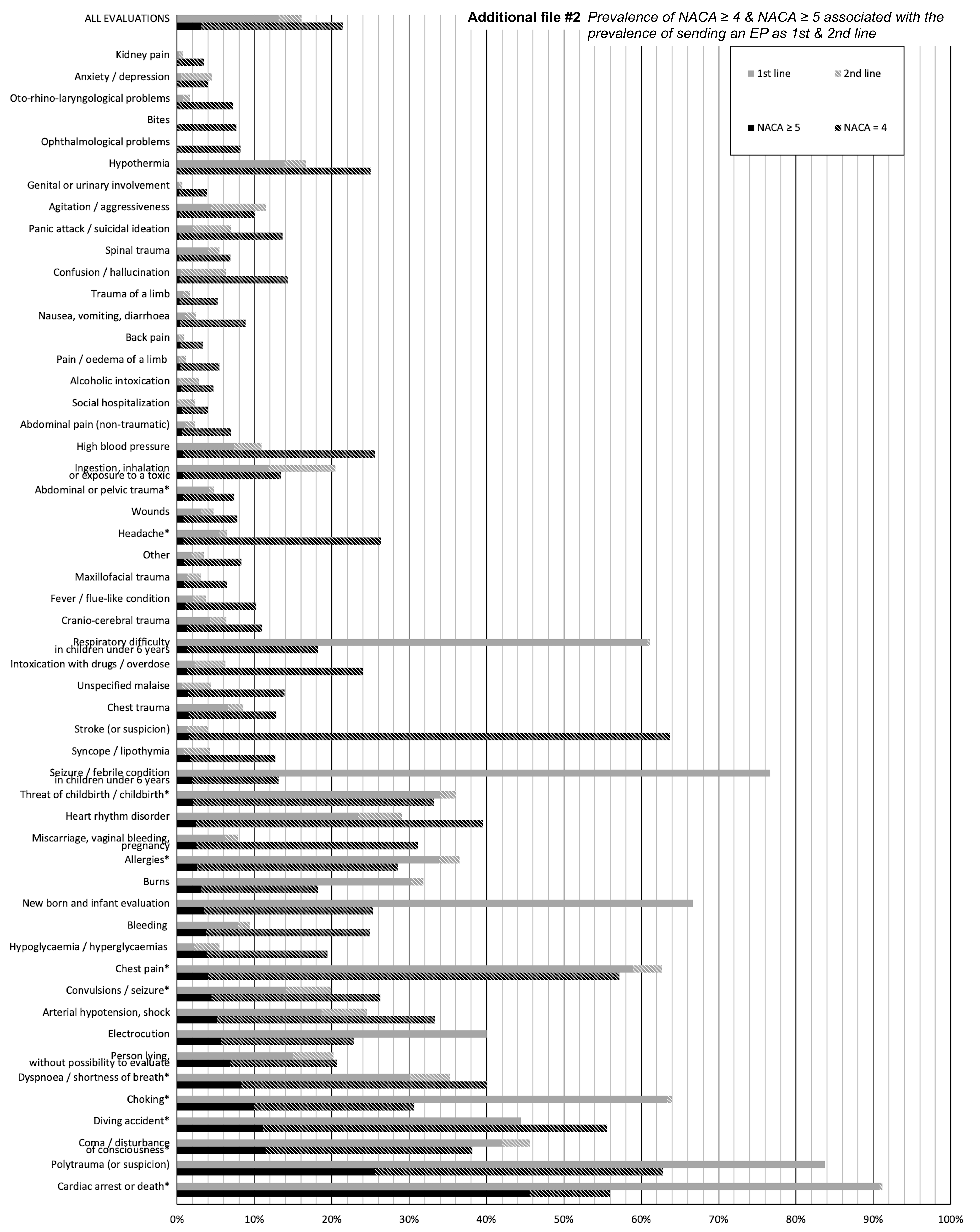

Supplement: Supplementary file 2 — Additional file 2 Prevalence of NACA ≥4 & NACA ≥5 associated with the prevalence of sending an EP as 1st & 2nd line. This figure links the prevalence of the priority dispatch of an EP (i.e. 1st-line and 2nd-line) with the prevalence of the NACA scale observed on site (i.e. RS-1 (NACA ≥4); RS-2 (NACA ≥5)) for each of the 53 symptoms. [file 13049_2021_844_MOESM2_ESM.png]
