# Supplementary material for: Emergency physician’s dispatch by a paramedic-staffed emergency medical communication centre: sensitivity, specificity and search for a reference standard
Source: Scand J Trauma Resusc Emerg Med. 2021 Feb 9;29:31. doi: 10.1186/s13049-021-00844-y (PMC7871575; doi:10.1186/s13049-021-00844-y)
Supplement: Supplementary file 3 — Additional file 3. “Diagnostic test” for each symptom using NACA ≥4 as reference standard. This table shows the “diagnostic test” applied for each of the 53 symptoms, thus detailing the sensitivity, specificity, positive & negative predictive values, over-triage & under-triage with their respective 95% confidence intervals for each symptom when using NACA ≥4 as reference standard. [file 13049_2021_844_MOESM3_ESM.pdf]

Additional file #3 *"Diagnostic test" for each symptom using NACA ≥ 4 as reference standard*

Symptoms	Reference Standard 1 (RS-1): NACA ≥ 4											
	Sensitivity [95% CI]		Specificity [95% CI]		PPV [95% CI]		NPV [95% CI]		Overtriage [95% CI]		Undertriage [95% CI]	
**ALL EVALUATIONS**	36.2%	[35.5% - 36.9%]	93.2%	[93% - 93.4%]	59.1%	[58.3% - 60%]	84.3%	[84% - 84.5%]	41%	[40% - 41.7%]	15.7%	[15.5% - 16%]
Kidney pain	0%	[0% - 18.5%]	99.8%	[98.9% - 100%]	0%	[0% - 97.5%]	96.5%	[94.5% - 97.9%]	100%	[2.5% - 100%]	3.5%	[2.1% - 5.5%]
Anxiety / depression	4.3%	[0.5% - 14.5%]	99.6%	[99.1% - 99.9%]	33.3%	[4.3% - 77.7%]	96.1%	[94.9% - 97.2%]	66.7%	[22.3% - 95.7%]	3.9%	[2.8% - 5.1%]
Oto-rhino-laryngological problems	0%	[0% - 18.5%]	99.1%	[96.9% - 99.9%]	0%	[0% - 84.2%]	92.7%	[88.7% - 95.6%]	100%	[15.8% - 100%]	7.3%	[4.4% - 11.3%]
Bites	0%	[0% - 97.5%]	100%	[73.5% - 100%]	0%	[0% - 24.7%]	92.3%	[64% - 99.8%]	100%	[75.3% - 100%]	7.7%	[0.2% - 36%]
Ophthalmological problems	0%	[0% - 52.2%]	100%	[93.6% - 100%]	-	-	91.8%	[81.9% - 97.3%]	-	-	8.2%	[2.7% - 18.1%]
Hypothermia	44.4%	[13.7% - 78.8%]	96.3%	[81% - 99.9%]	80%	[28.4% - 99.5%]	83.9%	[66.3% - 94.5%]	20%	[0.5% - 71.6%]	16.1%	[5.5% - 33.7%]
Genital or urinary involvement	2.5%	[0.1% - 13.2%]	99.9%	[99.4% - 100%]	50%	[1.3% - 98.7%]	96.2%	[94.9% - 97.3%]	50%	[1.3% - 98.7%]	3.8%	[2.7% - 5.1%]
Agitation / aggressiveness	15.2%	[10.5% - 21%]	96.9%	[95.9% - 97.6%]	35.3%	[25.2% - 46.4%]	91%	[89.6% - 92.3%]	64.7%	[53.6% - 74.8%]	9%	[7.7% - 10.4%]
Panic attack / suicidal ideation	10.2%	[6.7% - 14.5%]	99.1%	[98.6% - 99.5%]	65%	[48.3% - 79.4%]	87.5%	[85.9% - 89%]	35%	[20.6% - 51.7%]	12.5%	[11% - 14.1%]
Spinal trauma	29.4%	[21.6% - 38.1%]	97.8%	[97% - 98.5%]	50%	[38.1% - 61.9%]	94.9%	[93.8% - 95.9%]	50%	[38.1% - 61.9%]	5.1%	[4.1% - 6.2%]
Confusion / hallucination	1.7%	[0.5% - 4.2%]	99.7%	[99.2% - 99.9%]	44.4%	[13.7% - 78.8%]	85.9%	[84.1% - 87.5%]	55.6%	[21.2% - 86.3%]	14.1%	[12.5% - 15.9%]
Trauma of a limb	7.4%	[5.3% - 10%]	99.5%	[99.4% - 99.7%]	46.9%	[35.7% - 58.3%]	95.1%	[94.7% - 95.5%]	53.1%	[41.7% - 64.3%]	4.9%	[4.5% - 5.3%]
Nausea, vomiting, diarrhoea	0%	[0% - 3.4%]	98.9%	[98.1% - 99.4%]	0%	[0% - 26.5%]	91.1%	[89.3% - 92.6%]	100%	[73.5% - 100%]	8.9%	[7.4% - 10.7%]
Back pain	4.2%	[0.5% - 14.3%]	99.9%	[99.6% - 100%]	66.7%	[9.4% - 99.2%]	96.8%	[95.7% - 97.6%]	33.3%	[0.8% - 90.6%]	3.2%	[2.4% - 4.3%]
Pain / oedema of a limb	5.2%	[1.1% - 14.4%]	100%	[99.6% - 100%]	100%	[29.2% - 100%]	94.8%	[93.3% - 96%]	0%	[0% - 70.8%]	5.2%	[4% - 6.7%]
Alcoholic intoxication	0%	[0% - 2.8%]	99.8%	[99.5% - 99.9%]	0%	[0% - 45.9%]	95.3%	[94.4% - 96.1%]	100%	[54.1% - 100%]	4.7%	[3.9% - 5.6%]
Social hospitalization	0%	[0% - 26.5%]	100%	[98.7% - 100%]	-	-	96%	[93.1% - 97.9%]	-	-	4%	[2.1% - 6.9%]
Abdominal pain (non-traumatic)*	8.9%	[5.9% - 12.6%]	99.5%	[99.2% - 99.7%]	55.1%	[40.2% - 69.3%]	93.6%	[92.8% - 94.3%]	44.9%	[30.7% - 59.8%]	6.4%	[5.7% - 7.2%]
High blood pressure	15.7%	[11.3% - 21%]	95.5%	[93.7% - 96.9%]	54.4%	[41.9% - 66.5%]	76.7%	[73.7% - 79.5%]	45.6%	[33.5% - 58.1%]	23.3%	[20.5% - 26.3%]
Ingestion, inhalation or exposure to a toxic	41.2%	[18.4% - 67.1%]	92.7%	[86.2% - 96.8%]	46.7%	[21.3% - 73.4%]	91.1%	[84.2% - 95.6%]	53.3%	[26.6% - 78.7%]	8.9%	[4.4% - 15.8%]
Abdominal or pelvic trauma	39.5%	[28.8% - 51%]	98.7%	[97.8% - 99.3%]	71.1%	[55.7% - 83.6%]	95.3%	[93.9% - 96.5%]	28.9%	[16.4% - 44.3%]	4.7%	[3.5% - 6.1%]
Wounds	19.1%	[12.7% - 26.9%]	98.3%	[97.5% - 98.9%]	49%	[34.8% - 63.4%]	93.5%	[92.2% - 94.6%]	51%	[36.6% - 65.2%]	6.5%	[5.4% - 7.8%]
Headache*	9.2%	[5.8% - 13.5%]	95.8%	[94% - 97.2%]	44%	[30% - 58.7%]	74.7%	[71.6% - 77.6%]	56%	[41.3% - 70%]	25.3%	[22.4% - 28.4%]
Other	11.2%	[8.3% - 14.7%]	99%	[98.6% - 99.3%]	50%	[39.1% - 60.9%]	92.5%	[91.7% - 93.2%]	50%	[39.1% - 60.9%]	7.5%	[6.8% - 8.3%]
Maxillofacial trauma	5.6%	[2.3% - 11.3%]	98.9%	[98.4% - 99.4%]	26.9%	[11.6% - 47.8%]	93.9%	[92.7% - 94.9%]	73.1%	[52.2% - 88.4%]	6.1%	[5.1% - 7.3%]
Fever / flue-like condition	10%	[5.4% - 16.5%]	98.8%	[97.9% - 99.3%]	48.1%	[28.7% - 68.1%]	90.6%	[88.8% - 92.1%]	51.9%	[31.9% - 71.3%]	9.4%	[7.9% - 11.2%]
Cranio-cerebral trauma	14.5%	[11.8% - 17.5%]	96.9%	[96.4% - 97.4%]	36.9%	[30.8% - 43.3%]	90.2%	[89.4% - 91%]	63.1%	[56.7% - 69.2%]	9.8%	[9% - 10.6%]
Respiratory difficulty in children under 6 years	87.8%	[79.6% - 93.5%]	45.2%	[40.5% - 50%]	26.3%	[21.6% - 31.4%]	94.3%	[90.3% - 97%]	73.7%	[68.6% - 78.4%]	5.7%	[3% - 9.7%]
Intoxication with drugs / overdose	5.9%	[4.1% - 8.2%]	98.9%	[98.3% - 99.3%]	62.7%	[48.1% - 75.9%]	76.9%	[75% - 78.6%]	37.3%	[24.1% - 51.9%]	23.1%	[21.4% - 25%]
Unspecified malaise	2%	[1% - 3.5%]	99.5%	[99.3% - 99.7%]	41.4%	[23.5% - 61.1%]	86.3%	[85.2% - 87.3%]	58.6%	[38.9% - 76.5%]	13.7%	[12.7% - 14.8%]
Chest trauma	27.4%	[18.2% - 38.2%]	96.5%	[94.6% - 97.8%]	53.5%	[37.7% - 68.8%]	90%	[87.4% - 92.3%]	46.5%	[31.2% - 62.3%]	10%	[7.7% - 12.6%]
Stroke (or suspicion)	1.4%	[1% - 2%]	98.9%	[98.3% - 99.4%]	70.6%	[56.2% - 82.5%]	36.4%	[34.9% - 37.9%]	29.4%	[17.5% - 43.8%]	63.6%	[62.1% - 65.1%]
Syncope / lipothymia	1.2%	[0.4% - 2.7%]	99.2%	[98.8% - 99.5%]	17.2%	[5.8% - 35.8%]	87.3%	[86.1% - 88.4%]	82.8%	[64.2% - 94.2%]	12.7%	[11.6% - 13.9%]
Seizure / febrile condition in children under 6 years	93.5%	[82.1% - 98.6%]	20.5%	[16.6% - 24.9%]	16%	[11.8% - 20.9%]	96.3%	[89.7% - 99.2%]	84%	[79.1% - 88.2%]	3.7%	[0.8% - 10.3%]
Threat of childbirth / childbirth*	53.1%	[41.7% - 64.3%]	75.5%	[68.1% - 81.9%]	51.8%	[40.6% - 62.9%]	76.4%	[69.1% - 82.7%]	48.2%	[37.1% - 59.4%]	23.6%	[17.3% - 30.9%]
Heart rhythm disorder	42%	[38.7% - 45.3%]	88.8%	[87% - 90.4%]	71%	[66.9% - 74.8%]	70.1%	[67.9% - 72.3%]	29%	[25.2% - 33.1%]	29.9%	[27.7% - 32.1%]
Miscarriage, vaginal bleeding, pregnancy	14.7%	[7.6% - 24.7%]	97.6%	[93.9% - 99.3%]	73.3%	[44.9% - 92.2%]	71.7%	[65.3% - 77.5%]	26.7%	[7.8% - 55.1%]	28.3%	[22.5% - 34.7%]
Allergies*	60%	[53.7% - 66.1%]	76.6%	[73.1% - 79.8%]	50.5%	[44.7% - 56.3%]	82.8%	[79.5% - 85.7%]	49.5%	[43.7% - 55.3%]	17.2%	[14.3% - 20.5%]
Burns	58.3%	[36.6% - 77.9%]	75.9%	[66.7% - 83.6%]	35%	[20.6% - 51.7%]	89.1%	[80.9% - 94.7%]	65%	[48.3% - 79.4%]	10.9%	[5.3% - 19.1%]
New born and infant evaluation	77.3%	[54.6% - 92.2%]	36.9%	[25.3% - 49.8%]	29.3%	[18.1% - 42.7%]	82.8%	[64.2% - 94.2%]	70.7%	[57.3% - 81.9%]	17.2%	[5.8% - 35.8%]
Bleeding	22.7%	[19% - 26.9%]	97%	[96% - 97.8%]	71.5%	[63.4% - 78.7%]	79.1%	[77.1% - 81.1%]	28.5%	[21.3% - 36.6%]	20.9%	[18.9% - 22.9%]
Hypoglycaemia / hyperglycaemias	3.7%	[0.8% - 10.3%]	98.2%	[96.2% - 99.3%]	33.3%	[7.5% - 70.1%]	80.8%	[76.7% - 84.5%]	66.7%	[29.9% - 92.5%]	19.2%	[15.5% - 23.3%]
Chest pain*	71.6%	[70% - 73.1%]	57.8%	[55.9% - 59.7%]	69.3%	[67.8% - 70.9%]	60.4%	[58.4% - 62.3%]	30.7%	[29.1% - 32.2%]	39.6%	[37.7% - 41.6%]
Convulsions / seizure*	23.9%	[20.1% - 28%]	89.4%	[87.6% - 91%]	44.5%	[38.3% - 50.8%]	23.3%	[21.2% - 25.4%]	55.5%	[49.2% - 61.7%]	76.7%	[74.6% - 78.8%]
Arterial hypotension, shock	34.3%	[29% - 40%]	89.1%	[86.4% - 91.5%]	61.2%	[53.4% - 68.5%]	73.1%	[69.7% - 76.2%]	38.8%	[31.5% - 46.6%]	26.9%	[23.8% - 30.3%]
Electrocution	75%	[34.9% - 96.8%]	70.4%	[49.8% - 86.2%]	42.9%	[17.7% - 71.1%]	90.5%	[69.6% - 98.8%]	57.1%	[28.9% - 82.3%]	9.5%	[1.2% - 30.4%]
Person lying, without possibility to evaluate	35.7%	[32.5% - 39%]	90.3%	[89.3% - 91.3%]	49%	[45% - 52.9%]	84.4%	[83.2% - 85.6%]	51%	[47.1% - 55%]	15.6%	[14.4% - 16.8%]
Dyspnoea / shortness of breath*	48.8%	[47.1% - 50.4%]	82.4%	[81.4% - 83.4%]	64.9%	[63.1% - 66.7%]	70.7%	[69.5% - 71.8%]	35.1%	[33.3% - 36.9%]	29.3%	[28.2% - 30.5%]
Choking*	82.6%	[68.6% - 92.2%]	45.2%	[35.4% - 55.3%]	40%	[30.1% - 50.6%]	85.5%	[73.3% - 93.5%]	60%	[49.4% - 69.9%]	14.5%	[6.5% - 26.7%]
Diving accident*	60%	[14.7% - 94.7%]	75%	[19.4% - 99.4%]	75%	[19.4% - 99.4%]	60%	[14.7% - 94.7%]	25%	[0.6% - 80.6%]	40%	[5.3% - 85.3%]
Coma / disturbance of consciousness*	59.6%	[56.9% - 62.3%]	68.8%	[66.8% - 70.8%]	54.1%	[51.5% - 56.7%]	73.4%	[71.4% - 75.3%]	45.9%	[43.3% - 48.5%]	26.6%	[24.7% - 28.6%]
Polytrauma (or suspicion)	88.9%	[70.8% - 97.6%]	25%	[7.3% - 52.4%]	66.7%	[49% - 81.4%]	57.1%	[18.4% - 90.1%]	33.3%	[18.6% - 51%]	42.9%	[9.9% - 81.6%]
Cardiac arrest or death*	98.9%	[97.7% - 99.6%]	19.6%	[16% - 23.5%]	60.9%	[57.7% - 64.1%]	93.6%	[86.6% - 97.6%]	39.1%	[35.9% - 42.3%]	6.4%	[2.4% - 13.4%]
