# Supplementary material for: Emergency physician’s dispatch by a paramedic-staffed emergency medical communication centre: sensitivity, specificity and search for a reference standard
Source: Scand J Trauma Resusc Emerg Med. 2021 Feb 9;29:31. doi: 10.1186/s13049-021-00844-y (PMC7871575; doi:10.1186/s13049-021-00844-y)
Supplement: Supplementary file 4 — Additional file 4. “Diagnostic test” for each symptom using NACA ≥5 as reference standard. This table shows the “diagnostic test” applied for each of the 53 symptoms, thus detailing the sensitivity, specificity, positive & negative predictive values, over-triage & under-triage with their respective 95% confidence intervals for each symptom when using NACA ≥5 as reference standard. [file 13049_2021_844_MOESM4_ESM.pdf]

Additional file #4 *"Diagnostic test" for each symptom using NACA ≥ 5 as reference standard*

Symptoms	Reference Standard 2 (RS-2): NACA ≥ 5											
	Sensitivity [95% CI]		Specificity [95% CI]		PPV [95% CI]		NPV [95% CI]		Overtriage [95% CI]		Undertriage [95% CI]	
**ALL EVALUATIONS**	64.4%	[62.7% - 66.1%]	88.5%	[88.3% - 88.7%]	15.2%	[14.6% - 15.8%]	98.7%	[98.7% - 98.8%]	84.8%	[84.2% - 85.4%]	1.3%	[1.2% - 1.3%]
Kidney pain	-	-	99.8%	[98.9% - 100%]	0%	[0% - 97.5%]	100%	[99.3% - 100%]	100%	[2.5% - 100%]	0%	[0% - 0.7%]
Anxiety / depression	-	-	99.5%	[98.9% - 99.8%]	0%	[0% - 45.9%]	100%	[99.7% - 100%]	100%	[54.1% - 100%]	0%	[0% - 0.3%]
Oto-rhino-laryngological problems	-	-	99.2%	[97.1% - 99.9%]	0%	[0% - 84.2%]	100%	[98.5% - 100%]	100%	[15.8% - 100%]	0%	[0% - 1.5%]
Bites	-	-	100%	[75.3% - 100%]	-	-	100%	[75.3% - 100%]	-	-	0%	[0% - 24.7%]
Ophthalmological problems	-	-	100%	[94.1% - 100%]	-	-	100%	[94.1% - 100%]	-	-	0%	[0% - 5.9%]
Hypothermia	-	-	86.1%	[70.5% - 95.3%]	0%	[0% - 52.2%]	100%	[88.8% - 100%]	100%	[47.8% - 100%]	0%	[0% - 11.2%]
Genital or urinary involvement	0%	[0% - 84.2%]	99.8%	[99.3% - 100%]	0%	[0% - 84.2%]	99.8%	[99.3% - 100%]	100%	[15.8% - 100%]	0.2%	[0% - 0.7%]
Agitation / aggressiveness	0%	[0% - 52.2%]	95.6%	[94.6% - 96.5%]	0%	[0% - 4.2%]	99.7%	[99.4% - 99.9%]	100%	[95.8% - 100%]	0.3%	[0.1% - 0.6%]
Panic attack / suicidal ideation	40%	[5.3% - 85.3%]	98%	[97.2% - 98.6%]	5%	[0.6% - 16.9%]	99.8%	[99.5% - 100%]	95%	[83.1% - 99.4%]	0.2%	[0% - 0.5%]
Spinal trauma	40%	[5.3% - 85.3%]	96%	[95% - 96.9%]	2.7%	[0.3% - 9.4%]	99.8%	[99.5% - 100%]	97.3%	[90.6% - 99.7%]	0.2%	[0% - 0.5%]
Confusion / hallucination	0%	[0% - 52.2%]	99.5%	[99% - 99.8%]	0%	[0% - 33.6%]	99.7%	[99.3% - 99.9%]	100%	[66.4% - 100%]	0.3%	[0.1% - 0.7%]
Trauma of a limb	33.3%	[18% - 51.8%]	99.3%	[99.1% - 99.4%]	95.1%	[87.8% - 98.6%]	99.8%	[99.7% - 99.9%]	4.9%	[1.4% - 12.2%]	0.2%	[0.1% - 0.3%]
Nausea, vomiting, diarrhoea	0%	[0% - 52.2%]	99%	[98.3% - 99.5%]	0%	[0% - 26.5%]	99.6%	[99% - 99.9%]	100%	[73.5% - 100%]	0.4%	[0.1% - 1%]
Back pain	16.7%	[0.4% - 64.1%]	99.9%	[99.5% - 100%]	33.3%	[0.8% - 90.6%]	99.6%	[99.2% - 99.9%]	66.7%	[9.4% - 99.2%]	0.4%	[0.1% - 0.8%]
Pain / oedema of a limb	20%	[0.5% - 71.6%]	99.8%	[99.3% - 100%]	33.3%	[0.8% - 90.6%]	99.6%	[99% - 99.9%]	66.7%	[9.4% - 99.2%]	0.4%	[0.1% - 1%]
Alcoholic intoxication	0%	[0% - 21.8%]	99.8%	[99.5% - 99.9%]	0%	[0% - 45.9%]	99.5%	[99.1% - 99.7%]	100%	[54.1% - 100%]	0.5%	[0.3% - 0.9%]
Social hospitalization	0%	[0% - 84.2%]	100%	[98.8% - 100%]	-	-	99.3%	[97.6% - 99.9%]	-	-	0.7%	[0.1% - 2.4%]
Abdominal pain (non-traumatic)*	23.3%	[9.9% - 42.3%]	99%	[98.7% - 99.3%]	14.3%	[5.9% - 27.2%]	99.5%	[99.2% - 99.7%]	85.7%	[72.8% - 94.1%]	0.5%	[0.3% - 0.8%]
High blood pressure	28.6%	[70.95791% - 71%]	92.8%	[90.9% - 94.4%]	2.9%	[0.4% - 10.2%]	99.4%	[98.6% - 99.8%]	97.1%	[89.8% - 99.6%]	0.6%	[0.2% - 1.4%]
Ingestion, inhalation or exposure to a toxic	100%	[2.5% - 100%]	88.9%	[82.1% - 93.8%]	6.7%	[0.2% - 31.9%]	100%	[96.8% - 100%]	93.3%	[68.1% - 99.8%]	0%	[0% - 3.2%]
Abdominal or pelvic trauma	88.9%	[51.8% - 99.7%]	96.6%	[95.3% - 97.6%]	17.8%	[8% - 32.1%]	99.9%	[99.5% - 100%]	82.2%	[67.9% - 92%]	0.1%	[0% - 0.5%]
Wounds	64.3%	[35.1% - 87.2%]	97.5%	[96.6% - 98.2%]	17.6%	[8.4% - 30.9%]	99.7%	[99.3% - 99.9%]	82.4%	[69.1% - 91.6%]	0.3%	[0.1% - 0.7%]
Headache*	25%	[3.2% - 65.1%]	94.7%	[93% - 96.1%]	4%	[0.5% - 13.7%]	99.3%	[98.5% - 99.7%]	96%	[86.3% - 99.5%]	0.7%	[0.3% - 1.5%]
Other	31%	[17.6% - 47.1%]	98.4%	[98% - 98.7%]	14.8%	[8.1% - 23.9%]	99.4%	[99.1% - 99.6%]	85.2%	[76.1% - 91.9%]	0.6%	[0.4% - 0.9%]
Maxillofacial trauma	5.6%	[0.1% - 27.3%]	98.7%	[98.1% - 99.2%]	3.8%	[0.1% - 19.6%]	99.1%	[98.6% - 99.5%]	96.2%	[80.4% - 99.9%]	0.9%	[0.5% - 1.4%]
Fever / flue-like condition	28.6%	[8.4% - 58.1%]	98.2%	[97.3% - 98.8%]	14.8%	[4.2% - 33.7%]	99.2%	[98.5% - 99.6%]	85.2%	[66.3% - 95.8%]	0.8%	[0.4% - 1.5%]
Cranio-cerebral trauma	39.7%	[28.5% - 51.9%]	96.1%	[95.6% - 96.6%]	11.9%	[8.1% - 16.6%]	99.2%	[98.9% - 99.4%]	88.1%	[83.4% - 91.9%]	0.8%	[0.6% - 1.1%]
Respiratory difficulty in children under 6 years	100%	[59% - 100%]	39.7%	[35.5% - 44%]	2.1%	[0.9% - 4.4%]	100%	[98.3% - 100%]	97.9%	[95.6% - 99.1%]	0%	[0% - 1.7%]
Intoxication with drugs / overdose	30%	[14.7% - 49.4%]	98.1%	[97.5% - 98.6%]	17.6%	[8.4% - 30.9%]	99%	[98.5% - 99.4%]	82.4%	[69.1% - 91.6%]	1%	[0.6% - 1.5%]
Unspecified malaise	8.1%	[2.7% - 17.8%]	99.4%	[99.2% - 99.6%]	17.2%	[5.8% - 35.8%]	98.7%	[98.3% - 99%]	82.8%	[64.2% - 94.2%]	1.3%	[1% - 1.7%]
Chest trauma	70%	[34.8% - 93.3%]	94.4%	[92.3% - 96.1%]	16.3%	[6.8% - 30.7%]	99.5%	[98.6% - 99.9%]	83.7%	[69.3% - 93.2%]	0.5%	[0.1% - 1.4%]
Stroke (or suspicion)	8.3%	[2.8% - 18.4%]	98.8%	[98.4% - 99.1%]	9.8%	[3.3% - 21.4%]	98.6%	[98.1% - 98.9%]	90.2%	[78.6% - 96.7%]	1.4%	[1.1% - 1.9%]
Syncope / lipothymia	0%	[0% - 6.3%]	99.1%	[98.8% - 99.4%]	0%	[0% - 11.9%]	98.3%	[97.8% - 98.7%]	100%	[88.1% - 100%]	1.7%	[1.3% - 2.2%]
Seizure / febrile condition in children under 6 years	100%	[59% - 100%]	23.8%	[19.4% - 28.7%]	2.6%	[1.1% - 5.3%]	100%	[95.6% - 100%]	97.4%	[94.7% - 98.9%]	0%	[0% - 4.4%]
Threat of childbirth / childbirth*	60%	[14.7% - 94.7%]	66.5%	[60.2% - 72.5%]	3.6%	[0.8% - 10.2%]	98.8%	[95.6% - 99.8%]	96.4%	[89.8% - 99.2%]	1.2%	[0.2% - 4.4%]
Heart rhythm disorder	56.4%	[42.3% - 69.7%]	77.5%	[75.7% - 79.2%]	5.9%	[4.1% - 8.3%]	98.6%	[97.9% - 99.1%]	94.1%	[91.7% - 95.9%]	1.4%	[0.9% - 2.1%]
Miscarriage, vaginal bleeding, pregnancy	66.7%	[22.3% - 95.7%]	95.3%	[91.8% - 97.6%]	26.7%	[7.8% - 55.1%]	99.1%	[96.8% - 99.9%]	73.3%	[44.9% - 92.2%]	0.9%	[0.1% - 3.2%]
Allergies*	78.3%	[56.3% - 92.5%]	67.3%	[64.1% - 70.4%]	5.9%	[3.6% - 9.2%]	99.2%	[98% - 99.7%]	94.1%	[90.8% - 96.4%]	0.8%	[0.3% - 2%]
Burns	75%	[19.4% - 99.4%]	71.1%	[62.4% - 78.8%]	7.5%	[1.6% - 20.4%]	98.9%	[94.1% - 100%]	92.5%	[79.6% - 98.4%]	1.1%	[0% - 5.9%]
New born and infant evaluation	66.7%	[9.4% - 99.2%]	33.3%	[23.4% - 44.5%]	3.4%	[0.4% - 11.9%]	96.6%	[82.2% - 99.9%]	96.6%	[88.1% - 99.6%]	3.4%	[0.1% - 17.8%]
Bleeding	46.4%	[34.3% - 58.8%]	93.6%	[92.4% - 94.7%]	22.2%	[15.7% - 29.9%]	97.8%	[97% - 98.4%]	77.8%	[70.1% - 84.3%]	2.2%	[1.6% - 3%]
Hypoglycaemia / hyperglycaemias	0%	[0% - 20.6%]	97.8%	[95.8% - 99%]	0%	[0% - 33.6%]	96.1%	[93.8% - 97.8%]	100%	[66.4% - 100%]	3.9%	[2.2% - 6.2%]
Chest pain*	90.6%	[86.2% - 93.9%]	42.4%	[41.1% - 43.6%]	6.2%	[5.5% - 7.1%]	99.1%	[98.6% - 99.4%]	93.8%	[92.9% - 94.5%]	0.9%	[0.6% - 1.4%]
Convulsions / seizure*	35.8%	[25.4% - 47.2%]	86.9%	[85.2% - 88.5%]	11.3%	[7.7% - 15.9%]	96.7%	[95.7% - 97.5%]	88.7%	[84.1% - 92.3%]	3.3%	[2.5% - 4.3%]
Arterial hypotension. shock	48.9%	[34.1% - 63.9%]	82.9%	[80.3% - 85.4%]	13.5%	[8.8% - 19.6%]	96.8%	[95.2% - 97.9%]	86.5%	[80.4% - 91.2%]	3.2%	[2.1% - 4.8%]
Electrocution	100%	[15.8% - 100%]	63.6%	[45.1% - 79.6%]	14.3%	[1.8% - 42.8%]	100%	[83.9% - 100%]	85.7%	[57.2% - 98.2%]	0%	[0% - 16.1%]
Person lying. without possibility to evaluate	51.6%	[45.6% - 57.5%]	87.7%	[86.6% - 88.7%]	23.7%	[20.4% - 27.2%]	96.1%	[95.4% - 96.7%]	76.3%	[72.8% - 79.6%]	3.9%	[3.3% - 4.6%]
Dyspnoea / shortness of breath*	66.8%	[63.3% - 70.2%]	73.2%	[72.3% - 74.2%]	18.4%	[17% - 20%]	96.1%	[95.6% - 96.5%]	81.6%	[80% - 83%]	3.9%	[3.5% - 4.4%]
Choking*	93.3%	[68.1% - 99.8%]	40%	[31.7% - 48.8%]	14.7%	[8.3% - 23.5%]	98.2%	[90.3% - 100%]	85.3%	[76.5% - 91.7%]	1.8%	[0% - 9.7%]
Diving accident*	100%	[2.5% - 100%]	62.5%	[24.5% - 91.5%]	25%	[0.6% - 80.6%]	100%	[47.8% - 100%]	75%	[19.4% - 99.4%]	0%	[0% - 52.2%]
Coma / disturbance of consciousness*	77.6%	[73.2% - 81.6%]	62.6%	[60.8% - 64.3%]	21.1%	[19.1% - 23.3%]	95.6%	[94.6% - 96.4%]	78.9%	[76.7% - 80.9%]	4.4%	[3.6% - 5.4%]
Polytrauma (or suspicion)	90.9%	[58.7% - 99.8%]	18.8%	[7.2% - 36.4%]	27.8%	[14.2% - 45.2%]	85.7%	[42.1% - 99.6%]	72.2%	[54.8% - 85.8%]	14.3%	[0.4% - 57.9%]
Cardiac arrest or death*	99.1%	[97.8% - 99.8%]	16.2%	[13.2% - 19.5%]	49.7%	[46.5% - 53%]	95.7%	[89.5% - 98.8%]	50.3%	[47% - 53.5%]	4.3%	[1.2% - 10.5%]
